# Giant Cell Arteritis With Bilateral Central Retinal Artery Occlusion and Tongue Necrosis

**DOI:** 10.7759/cureus.59554

**Published:** 2024-05-02

**Authors:** Anand S Brar, Ridham Nanda, Raja Narayanan, Srikanta K Padhy

**Affiliations:** 1 Ophthalmology, LV Prasad Eye Institute, Mithu Tulsi Chanrai (MTC) Campus, Bhubaneswar, IND; 2 Ophthalmology, All India Institute of Medical Sciences, Vijaypur, Jammu, Jammu, IND; 3 Vitreoretinal Diseases, LV Prasad Eye Institute, Hyderabad, IND

**Keywords:** ischemic stroke, temporal artery biopsy, tongue necrosis, central retinal artery occlusion, giant cell arteritis

## Abstract

This case report describes a complicated case of giant cell arteritis (GCA) with tongue necrosis and bilateral central retinal artery occlusion (CRAO). An 81-year-old male patient with a history of recent retinal artery occlusion, ischemic stroke, and hypertensive emergency was evaluated. Clinical examination, including a visual acuity assessment, fundus evaluation, and oral examination, was performed. Laboratory investigations, such as erythrocyte sedimentation rate (ESR), were conducted. A temporal artery biopsy was performed to confirm the diagnosis of GCA. The patient presented with sudden vision loss in the left eye following a prior episode of retinal artery occlusion in the right eye. Ophthalmoscopic examination revealed CRAO in the left eye. Additionally, tongue necrosis, a rare manifestation of GCA, was observed. The ESR was significantly elevated. A temporal artery biopsy supported the diagnosis of GCA. The patient was promptly referred for immunologist consultation and initiated on intravenous methylprednisolone therapy. This case highlights the diverse and potentially devastating nature of GCA, involving ocular and systemic manifestations. Bilateral CRAO and tongue necrosis are rare but significant complications of GCA. Prompt diagnosis and early initiation of corticosteroid therapy are crucial to prevent irreversible visual loss and further complications. A multidisciplinary approach involving ophthalmologists and other specialists is essential for the comprehensive management of GCA.

## Introduction

Giant cell arteritis (GCA) is an immune-mediated vasculitis that affects medium and large arteries that have an elastic lamina inside them [1-3]. This disease primarily affects the temporal arteries along with other extracranial branches of the carotid artery. While GCA is more prevalent among Caucasians, it has also been reported in non-Caucasian populations worldwide, including in India, where it is often underdiagnosed [2]. GCA primarily affects individuals over the age of 50, with women being three times more susceptible than men [3]. The disease presents a wide range of systemic symptoms and signs, making diagnosis challenging. The most common symptom of GCA includes a new-onset headache, which is characteristically stabbing in nature and temporal in location. It occurs due to the stimulation of sensory nerve fibers within inflamed temporal arteries [3]. Common presentations include scalp tenderness, jaw claudication, myalgia, transient ischemic attacks, and tender nodular and tender temporal arteries [3]. Systemic presentation usually precedes ophthalmic manifestations. Ophthalmic involvement in GCA, particularly visual loss, is a severe complication and an ophthalmic emergency [2]. Temporal artery biopsy (TAB) is considered the gold standard for diagnosis, although false-negative results can occur [3].

Systemic corticosteroids are the treatment of choice in patients diagnosed with GCA. Corticosteroids are administered in various modalities, including intravenous and oral. Different dosing schedules for GCA have been described, but patients with ocular or cardiovascular complications usually require higher doses of steroids. Therapy is initiated with a three-day dosing of intravenous methylprednisolone (1 g/day), followed by a high oral dosing of prednisolone (80-100 mg/day), gradually tapered over 6-8 months [3]. Subsequently, the patient may require low-dose steroids or immunosuppressive agents to maintain remission of the disease for 1-2 years. The treatment protocol for each patient needs to be customized as per monitoring of the erythrocyte sedimentation rate (ESR) and reporting of side effects of steroids. Various immunomodulatory drugs like methotrexate, cyclophosphamide, cyclosporine, and azathioprine have shown variable results in GCA and may be used to reduce steroid dependence [3].

This case highlights the diverse nature of GCA and the importance of early recognition and multidisciplinary management.

## Case presentation

**Figure 1:**
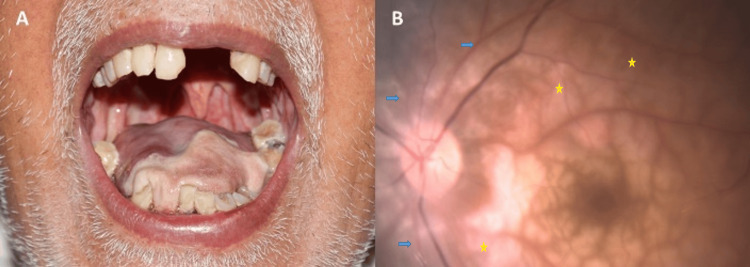
(A) Clinical photograph demonstrating tongue necrosis in the patient with giant cell arteritis. (B) Color fundus photograph of the left eye revealing attenuated arteries (blue arrows) and posterior pole opacification (yellow star marks), indicative of central retinal artery occlusion in the patient.

We present the case of an 81-year-old male who urgently presented to the Ophthalmology emergency department with a sudden, painless loss of vision in his left eye. This event occurred approximately two weeks following a central retinal artery occlusion (CRAO) in his right eye. An ophthalmologist was consulted at this episode, and various systemic investigations were advised to the patient, but one day later the patient's condition suddenly deteriorated as he had a hypertensive emergency and loss of consciousness. He was rushed to the hospital; his blood pressure was recorded to be 260/120 mm Hg; baseline systemic investigations were done. His lipid profile was abnormal, with strikingly raised cholesterol (450 mg/dl), ESR (72 mm/hour), and a normal total leucocyte count of 8000 cells/ml. His renal function tests were normal with mildly elevated liver enzymes (SGOT-95 units/L, SGPT-129 units/L). The patient was diagnosed with an ischemic stroke involving the cerebellum (confirmed on a computed tomography scan of the head) that necessitated admission to the intensive care unit (ICU). He was treated with anti-hypertensives, lipid-lowering agents, and anticoagulants. One week later, he was discharged from the ICU and referred to our clinic. On examination, he exhibited neurological symptoms such as difficulty swallowing, sialorrhea, and an unsteady gait. Remarkably, despite these challenges, the patient remained alert and oriented. The assessment of visual acuity revealed a complete absence of light perception in the right eye, while the left eye was limited to counting fingers at one meter. Dilated fundus evaluation unveiled a CRAO in the left eye (Figure 1). A comprehensive oral examination further revealed the presence of tongue necrosis (Figure 1), suggestive of involvement of the lingual artery. As the patient's ESR was found to be significantly elevated at 72 mm/hour and he had bilateral CRAO, we suspected GCA. To confirm the diagnosis, a TAB was performed, providing compelling evidence supporting the presence of GCA. The patient was urgently referred to an immunologist for further management, where intravenous methylprednisolone was initiated, and later he was advised oral systemic steroids (prednisolone). Antihypertensives and lipid-lowering agents were continued. At the one-month follow-up, the visual acuity remained the same, with the fundus revealing resolving retinal edema. The patient had improvements in overall general condition, including neurological symptoms.

## Discussion

GCA is the most prevalent systemic vasculitis in adults, characterized by inflammation of medium- to large-sized arteries [3]. The disease exhibits a broad range of systemic manifestations, necessitating a multidisciplinary approach to its diagnosis and management. Ocular involvement in GCA can result in various visual complications, including anterior ischemic optic neuropathy (A-AION), cilioretinal artery occlusion, CRAO, and arteritic posterior ischemic optic neuropathy (A-PION) [2]. In this case report, we present an elderly male patient with a complex medical history, including a recent CRAO, ischemic stroke, and hypertensive emergency. Notably, the patient presented with a unique combination of ocular complications, lingual artery involvement, and associated systemic symptoms, highlighting the diverse and potentially devastating nature of GCA.

The ocular manifestations of GCA are well documented, with CRAO occurring in 2% to 18% of cases [2]. In our patient, the sudden and painless loss of vision in the left eye following a prior retinal artery occlusion in the other eye raised suspicion for GCA-related vascular compromise. Furthermore, the coexistence of bilateral visual loss at presentation or within a few weeks has been reported in 28% to 31% of GCA patients [2]. This emphasizes the importance of considering GCA as an underlying etiology in individuals presenting with rapid and sequential retinal arterial occlusion. GCA demonstrates a unique preference for the posterior ciliary arteries (PCAs) among the orbital arteries. These PCAs supply the choroid, optic nerve head, and cilioretinal artery. The ocular manifestations of GCA primarily arise from ischemic processes caused by thrombotic occlusion resulting from granulomatous inflammation affecting the PCAs. In contrast, involvement of the ophthalmic artery in GCA is rare compared to the predominant affection of the PCAs [2,3]. The central retinal artery and one of the PCAs often share a common trunk. When individuals with an age greater than 50 years present with CRAO, fluorescein fundus angiography should be performed to assess for underlying PCA occlusion. The presence of PCA occlusion is highly suggestive of GCA-related CRAO [2-4]. Unfortunately, fluorescein fundus angiography could not be performed in our case due to the patient's poor systemic condition.

Previous treating physicians did not consider GCA as a differential diagnosis, given the possibility of a hypertensive crisis ultimately leading to stroke. The patient also had hyperlipidemia, which can cause arteriosclerotic disease and subsequent transient ischemic attacks and strokes, along with central retinal artery occlusion. GCA must be differentiated from other closely related connective tissue disorders like polymyalgia rheumatica, systemic lupus erythematosus, polyarteritis nodosa, polymyositis, and Takayasu arteritis [3]. Characteristic temporal artery tenderness and a temporal artery biopsy help establish the diagnosis. Decreased vision in GCA must be differentiated from other causes of non-arteritic-AION, including diabetes, hypertension, and disc overcrowding [2,3]. Such patients don’t report symptoms of headache, tenderness over the temporal artery, or jaw claudication; the ESR is usually in the normal range.

Beyond ocular involvement, our patient exhibited lingual artery affection, as evidenced by tongue necrosis, which is an exceedingly rare manifestation of GCA. Tongue necrosis has been reported in fewer than 70 cases over the past five decades, further highlighting its rarity [5]. Bilateral lingual artery necrosis, as observed in our patient, is an even more exceptional finding, with only a handful of cases reported in the literature [5-8]. This severe ischemic insult to the tongue necessitates urgent evaluation and treatment to prevent further complications and associated morbidity. Given the rarity of lingual artery involvement in GCA, the recognition of this atypical presentation is crucial for timely diagnosis and intervention.

In this case, the erythrocyte sedimentation rate (ESR) was significantly elevated, aligning with the typical laboratory findings of GCA. ESR measurement is a crucial diagnostic tool, aiding in the evaluation of disease activity and response to treatment [3]. The diagnosis of GCA is challenging but essential, as prompt initiation of systemic corticosteroid therapy can prevent irreversible complications [3,9]. Systemic steroids and immunosuppressive are the main stay of treatment in GCA [9].

## Conclusions

This case report highlights the significance of a comprehensive and multidisciplinary approach in the diagnosis and management of GCA. Ophthalmologists play a pivotal role in recognizing ocular complications, such as CRAO, and should remain vigilant for associated systemic involvement. Furthermore, the presence of atypical manifestations, such as tongue necrosis, warrants early consideration of GCA to prevent delays in diagnosis and treatment initiation.
